# Dysregulation of locus coeruleus development in congenital central hypoventilation syndrome

**DOI:** 10.1007/s00401-015-1441-0

**Published:** 2015-05-15

**Authors:** Hiroko Nobuta, Maria Roberta Cilio, Olivier Danhaive, Hui-Hsin Tsai, Srinivasan Tupal, Sandra M. Chang, Alice Murnen, Faith Kreitzer, Verenice Bravo, Catherine Czeisler, Hamza Numan Gokozan, Patrick Gygli, Sean Bush, Debra E. Weese-Mayer, Bruce Conklin, Siu-Pok Yee, Eric J. Huang, Paul A. Gray, David Rowitch, José Javier Otero

**Affiliations:** Department of Pediatrics, University of California San Francisco, 35 Medical Center Way, San Francisco, CA 94143 USA; Department of Neurology, University of California San Francisco, San Francisco, 35 Medical Center Way, CA 94143 USA; Department of Medicine, University of California San Francisco, 35 Medical Center Way, San Francisco, CA 94143 USA; Department of Pathology, University of California San Francisco, 35 Medical Center Way, San Francisco, CA 94143 USA; Department of Howard Hughes Medical Institute, University of California San Francisco, 35 Medical Center Way, San Francisco, CA 94143 USA; Department of Anatomy and Neurobiology, Washington University School of Medicine, 660 S. Euclid Ave, St. Louis, MO 63110 USA; Gladstone Institutes, 1650 Owens Street, San Francisco, CA 94158 USA; Department of Genetics and Developmental Biology, University of Connecticut Health Center, 263 Farmington Ave, Farmington, CT 06030-3001 USA; Department of Pathology, The Ohio State University College of Medicine, 4169 Graves Hall, 333 W 10th Ave, Columbus, OH 43210 USA; Center for Autonomic Medicine in Pediatrics (CAMP), Ann and Robert H. Lurie Children’s Hospital of Chicago and Stanley Manne Children’s Research Institute, Northwestern University Feinberg School of Medicine, 225 East Chicago Avenue, Box 165, Chicago, IL 60611-2605 USA

**Keywords:** Congenital central hypoventilation syndrome, Locus coeruleus, Noradrenergic system, PHOX2B

## Abstract

Human congenital central hypoventilation syndrome (CCHS), resulting from mutations in transcription factor *PHOX2B,* manifests with impaired responses to hypoxemia and hypercapnia especially during sleep. To identify brainstem structures developmentally affected in CCHS, we analyzed two postmortem neonatal-lethal cases with confirmed polyalanine repeat expansion (PARM) or Non-PARM (*PHOX2B∆8*) mutation of *PHOX2B.* Both human cases showed neuronal losses within the locus coeruleus (LC), which is important for central noradrenergic signaling. Using a conditionally active transgenic mouse model of the *PHOX2B∆8* mutation, we found that early embryonic expression (<E10.5) caused failure of LC neuronal specification and perinatal respiratory lethality. In contrast, later onset (E11.5) of *PHOX2B∆8* expression was not deleterious to LC development and perinatal respiratory lethality was rescued, despite failure of chemosensor retrotrapezoid nucleus formation. Our findings indicate that early-onset mutant *PHOX2B* expression inhibits LC neuronal development in CCHS. They further suggest that such mutations result in dysregulation of central noradrenergic signaling, and therefore, potential for early pharmacologic intervention in humans with CCHS.

**Electronic supplementary material:**

The online version of this article (doi:10.1007/s00401-015-1441-0) contains supplementary material, which is available to authorized users.

## Introduction

Congenital central hypoventilation syndrome (CCHS) is a classic disorder of autonomic respiratory control characterized by alveolar hypoventilation and monotonous respiratory rates despite abnormal pCO_2_ and pH concentrations, especially during non-rapid eye movement (NREM) sleep [57]. Patients with CCHS lack behavioral responsiveness to hypoxemia and hypercarbia without symptoms of shortness of breath or respiratory distress [8], and will not automatically adjust spontaneous ventilation or awaken from sleep despite progressive physiologic compromise [57]. A subset of CCHS patients have Hirschsprung disease (HSCR; absence of ganglion cells from variable lengths of distal bowel), and/or solid extracranial tumors of neural crest origin [6, 53], and additional symptoms of autonomic nervous system dysregulation are reported [18, 20, 42, 48].

Autonomic respiratory networks are stimulated when specialized neuronal sensors (chemosensors) detect low levels of O_2_ and/or high levels of CO_2_ in the blood. These chemosensors include the carotid bodies (CB), located in the peripheral nervous system (PNS) near the bifurcation of the carotid artery, and several central nervous system (CNS) nuclei [22]. Specialized neurons and astrocytic populations in brain stem contribute to central CO_2_ chemosensation [9, 19]. Classical pharmacological studies show that catecholaminergic neuron depletion in the brain stem results in decreased ventilatory response to elevated CO_2_ levels [31]. The rodent retrotrapezoid nucleus (RTN), located ventral to the facial nerve nucleus, drives respiration in response to decreased pH, resulting from elevated blood CO_2_ concentrations [34]. Interestingly, such hypercapnic ventilatory responses were diminished in adult rodents after chemical ablation of the locus coeruleus (LC) [7], a major central noradrenergic structure that is located in dorsal brainstem with extensive connections to other local nuclei as well as forebrain.

**Fig. 1:**
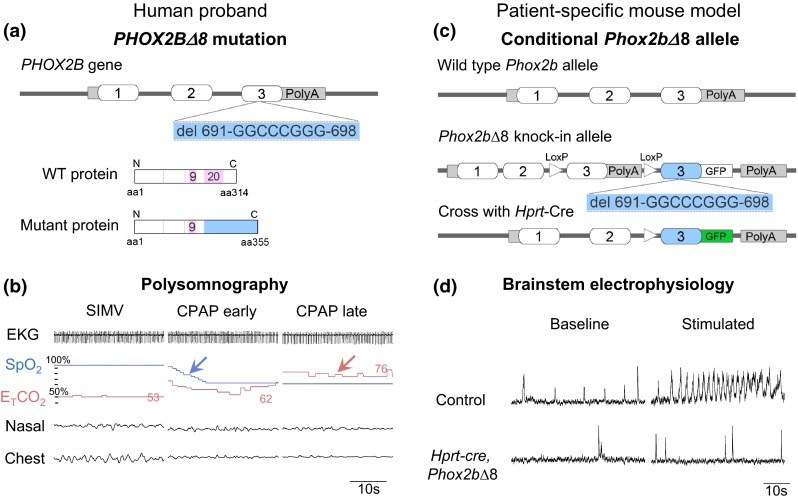
Genotype and respiratory physiological phenotypes of the proband and transgenic mouse model. **a** *PHOX2B* contains three exons and proband 8-nucleotide frameshift mutation in exon 3 is shown. Nucleotide number refers to nucleotide position of GenBank accession number CCDS 3463.1. Wild-type PHOX2B protein is 314 amino acids long; *PHOX2B∆8* mutation results in loss of the 20-alanine repeat domain and generates a protein of 355 amino acids. **b** Polysomnographic recording from the proband while on Synchronized Intermittent Mandatory Ventilation (SIMV) and after switching to continuous positive airway pressure (CPAP). Note the rapid decrease in oxygen saturation (*blue arrow*) and increase in carbon dioxide (*pink arrow*) levels. Values of E_T_CO_2_ at each epoch shown. When challenged with persistent hypercarbia and hypoxemia during CPAP, the proband showed no increase in respiratory effort. Heart rate variability during the challenge was minimal. *EKG* electrocardiogram, *SpO*_*2*_ oxygen saturation, *E*_*T*_ *CO*_*2*_ end-tidal CO_2_, *Nasal* nasal airflow, *Chest* chest wall movements from respiratory inductance plethysmography. CPAP pressure of 5 cm H_2_O was used. SIMV rate was 45 breaths/min. “Early” refers to 45 s after switching to CPAP, “late” refers to 75 s after switching to CPAP. Time scale is shown. **c** Targeting construct of patient-specific mouse model. Human *PHOX2B* exon 3 containing patient-specific *PHOX2B* mutation (denoted in *blue color*) is inserted following unmodified, non-mutated mouse *Phox2b* exon 3 flanked by loxP sites, to allow conditional expression of mutant gene by cre recombinase. For detailed generation of transgenic mouse line see Figure S4. **d** Endogenous respiratory output. Integrated C4 inspiratory activity from E18.5 control and *Hprt*-*cre*, *Phox2b∆8* mutant mice under baseline (*left*) and stimulated (1 μM substance P, *right*) conditions. Note lack of response in *Phox2b∆8* mouse brainstem (*n* = 4, a representative recording shown)

The human paired-like homeodomain transcription factor *PHOX2B* contains C-terminal 9- and 20-alanine repeats (Fig. 1a). Heterozygous mutations of *PHOX2B* are etiologic in CCHS [1, 59], comprising polyalanine repeat expansion mutations (PARMs; 90–92 % of cases) in exon 3, as well as non-PARMs with missense, frameshift, nonsense and stop-codon mutations (NPARMs; 8–10 % of cases) throughout the coding region, and whole- or partial-gene deletions (<1 % of cases) [27]. A genotype–phenotype correlation has been shown between *PHOX2B* polyalanine repeat length and severity of the respiratory phenotype, associated symptoms, and the age of onset [2, 33, 58, 59]. Large heterozygous NPARM deletions within exon 3 are correlated with the most severe CCHS phenotype with complete apnea and/or profound hypoventilation during sleep, severe hypoventilation during wakefulness, and intestinal aganglionosis from duodenum to anus [6, 33, 53].

In rodents, RTN development requires *Phox2b* function [12], and mouse models of CCHS, either expressing the 27-polyalanine repeat PARM or NPARM mutations of *Phox2b,* prevent RTN formation [13, 35]. Although *Phox2b* is a well-known regulator of motor and noradrenergic neuronal specification [39, 40], the precise basis of respiratory control in CCHS remains incompletely understood. Indeed, abnormal development or injury to central noradrenergic structures is suggested by prior work. The LC is the major source of central noradrenergic signaling [5]; it is thus a major regulator of arousal state and is also thought to function in cognition [49]. Several lines of evidence support the role of LC as a central chemosensor [11, 17], but owing to the lack of neuropathological information from CCHS patients with confirmed *PHOX2B* mutations, it remains unclear whether CCHS-associated *PHOX2B* mutations primarily affect LC development.

To achieve further insight into the pathobiology of CCHS, we analyzed two postmortem cases of neonatal-lethal CCHS with confirmed *PHOX2B* mutations. Proband 1 was a full-term neonate with a heterozygous NPARM deletion/frameshift mutation (*PHOX2B∆8*), resulting in severe hypoventilation and total intestinal aganglionosis. Proband 2 was born preterm and had the most common heterozygous CCHS *PHOX2B* 20/27 mutation (PARM) with less severe phenotype. Interestingly, both cases showed loss or severe diminishment of noradrenergic LC neurons. We modeled the NPARM case in vivo by generating a cognate conditional transgenic mouse line. Early embryonic conditional activation of *Phox2b∆8* in mouse brainstem (<E10.5) caused loss of a functional LC and abnormalities in all central noradrenergic (NA) neurons (e.g., A1/C2, forebrain projections to hypothalamus) tested. In contrast, later-onset (E11.5) activation of *Phox2b∆8* expression spared NA neuron development and was not perinatal respiratory-lethal, despite loss of the RTN. Our findings demonstrate that LC development is compromised, and suggest abnormal central noradrenergic signaling, as a component of human CCHS.

## Materials and methods

### Human neuropathological studies

Postmortem human samples (proband 1) were obtained using University of California, San Francisco (UCSF) guidelines with oversight of the Committee for Human Research and Gamete, Embryo and Stem Cell Research (GESCR) committee. Tissue from *PHOX2B 20/27* proband 2 was obtained from Rainbow Babies and Children’s Hospital (Cleveland, OH, USA) under an IRB-approved protocol at The Ohio State University. The entire formalin-fixed brainstems were serially sectioned for microscopic evaluation and compared to samples obtained from four roughly age-matched (control) patients that expired from other diseases. For case histories of controls, histological and immunohistochemical (IHC) procedures, see Suppl. Data.

### *Phox2b∆8* mouse model generation and animal husbandry

We generated a transgenic mouse line carrying a cre–loxP-inducible allele of human *PHOX2B∆8* exon 3 by BAC recombineering and homologous recombination in embryonic stem cells (ES cells; See Suppl. Data). For early-onset (germline) activation of *Phox2b∆8* allele, *Hprt*-*cre* mice (JAX 004302 on C57/Bl6 background) were intercrossed to *Phox2b∆8* heterozygotes. For late-onset CNS activation of *Phox2b∆8* allele, we used *Blbp*-*cre* [23]. Mutant mice in each genotype were compared to cre-negative littermate controls. Animal procedures were approved by Institutional Animal Care and Use Committees at Washington University (St. Louis, MO, USA), University of Connecticut Health Center (Farmington, CT, USA) and UCSF (San Francisco, CA, USA).

### Mouse tissue processing, and histology

Embryos were collected from time-pregnant females (E0.5 at time of plug recognition) under deep anesthesia; those older than E16.5 were perfused with PBS followed by 4 % PFA under hypothermia anesthesia. For IHC, tissues were post-fixed in 4 % PFA and cryoprotected, frozen in OCT and sectioned at 14 μm. Cryosections were subjected to antigen retrieval in citrate buffer, pH 6.0 for 10 min at 90 °C as necessary, blocked with 5 % donkey serum in PBS with 0.3 % Triton X-100, incubated with primary antibodies overnight at 4 °C, followed by appropriate secondary antibodies (see Suppl. Data) for 1 h at room temperature prior to imaging on a Nikon 80i microscope equipped with Hamamatsu CCD camera.

### In vitro mouse explant respiratory physiology

In vitro explant respiratory physiology was performed as described [26]. Brainstem–spinal (en bloc) preparations with an anterior transection near diencephalon–midbrain junction were made using E18.5 embryos as detailed in Suppl. Data.

### Quantifications and statistical analysis

All statistical analysis was performed using Microsoft Excel (Mac Office 2008) or R version 2.11.1. Transgenic mouse phenotypes were analyzed with Student’s *t* test.

## Results

### Human CCHS proband 1: clinical history and neuropathological findings

A full-term male presented with respiratory depression at birth, apnea and oxygen desaturation that required mechanical ventilation. Polysomnography demonstrated normal baseline oxygen saturation (SpO_2_) and end-tidal CO_2_ (E_T_CO_2_) while mechanically ventilated (Fig. 1b, Figure S1). However, when challenged by removal of the ventilator-generated respiratory rate, proband 1 showed hypoventilation resulting in oxygen desaturation (nadir 57 %) and rise of E_T_CO_2_ (peak 82 mmHg) during both wakefulness and sleep. Persistent and profound hypoxemia and hypercapnia failed to induce chemoreceptor reflexes (i.e., increased breathing rate/effort or a variation of heart rate); there was no arousal response from sleep (Fig. 1b, Figure S1). Magnetic resonance imaging and spectroscopy of the brain were normal. The electroencephalogram showed normal brain activity, both during wakefulness and sleep, without seizures. Proband 1 had permanently dilated pupils with non-measurable response to light, suggesting autonomic dysfunction. Because of enteral feeding intolerance, he was dependent on total parenteral nutrition. The intestine showed pervasive aganglionosis from 10 cm distal to the ligament of Treitz to the rectum (Figure S2), indicating HSCR disease.

Genomic DNA analysis demonstrated an eight-nucleotide deletion in exon 3 of *PHOX2B* (Fig. 1a; cDNA position 691-GGCCCGGG-698; heretofore called *PHOX2B∆8*). This caused a frameshift that removed the alanine repeat-generated elongated aberrant residues from amino acid 230 to the C-terminus (Figure S3a). Maternal DNA showed intact copies of *PHOX2B*; however, our analysis does not rule out possible low-level somatic mosaicism [28]. Paternal DNA was unavailable. Together, these clinical, genetic, and pathological findings confirmed a NPARM *PHOX2B* mutation and diagnosis of CCHS with intestinal aganglionosis. Following withdrawal of life support at 6 weeks of age, an autopsy was performed.

**Fig. 2:**
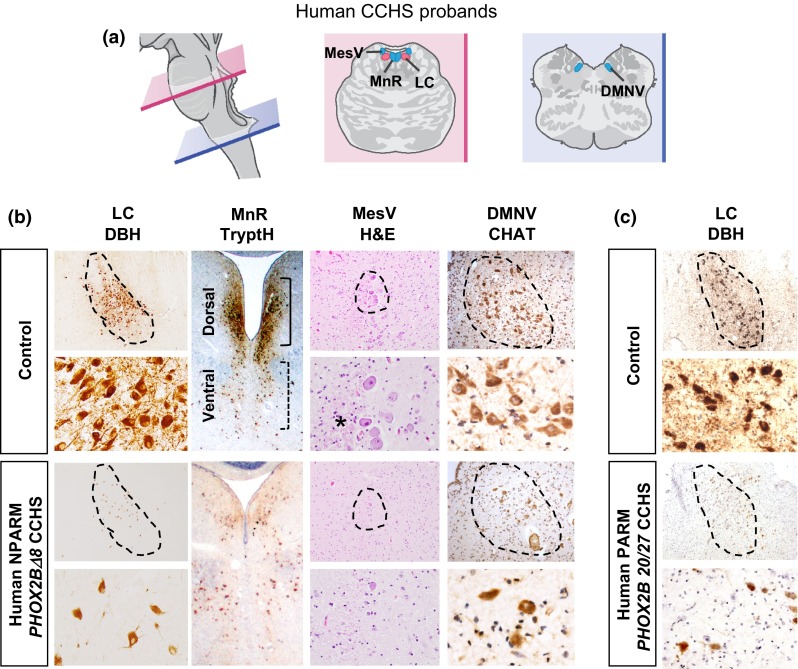
Brainstem pathology in CCHS probands. **a** Cartoon of human hindbrain at levels of pons (*pink*) and medulla (*blue*) is shown. **b** Dramatic losses were observed in NPARM *PHOX2BΔ8* proband locus coeruleus (LC), dorsal median raphe (MnR), mesencephalic trigeminal nucleus (MesV) and dorsal motor nucleus of vagus (DMNV). Note lack of dopamine β-hydroxylase (DBH) expression in LC and tryptophan hydroxylase (TrypH) in dorsal MnR indicating defects in synthesis of noradrenaline and serotonin production. DMNV showed diminished cholinergic neurons, indicated by choline acetyltransferase (CHAT) expression. *Asterisk* indicates diminished MesV fibers originating from the nucleus in the proband. *H&E* hematoxylin and eosin. **c** Dramatic loss was observed in PARM *PHOX2B* 20/27 proband LC. Note a significant reduction of DBH expression

Postmortem examination showed a dramatic loss of LC neurons that express dopamine β-hydroxylase (DBH) (Fig. 2a, b). The dorsal median raphe (dMnR), a major source of central serotonergic innervation, was severely diminished. We found additional losses of the hindbrain mesencephalic trigeminal nucleus (MesV) and dorsal motor nucleus of the vagus (DMNV), which derive from Phox2b+ progenitors [12, 24] (Fig. 2a, b; Table S1). In contrast, there were no gross or microscopic abnormalities detectable in cerebral cortex, striatum or thalamus (not shown), or significant abnormalities of the medullary arcuate nucleus, CNVII (facial nucleus), or surrounding areas including inferior olive, area postrema or nucleus prepositus (Figure S3b).

### Dysregulated brain stem development in *PHOX2B∆8* conditional mouse model

To assess function of *PHOX2B∆8* during development in vivo, we generated a conditional transgenic mouse line by targeted homologous recombination in ES cells (Fig. 1c). Note because human and mouse exon 3 are identical at the amino acid level we used mutant human exon 3 for conditional expression of *PHOX2B∆8* in mouse. In this line, the engineered *PHOX2B∆8* allele is activated by bacteriophage P1 *cre* recombinase, which initiates expression of PHOX2B∆8 proteins and a downstream green fluorescent protein (GFP) reporter gene, as shown in Figure S4a.

We crossed this line with the germline driver *Hprt*-*cre* to activate recombination in all tissues from early embryonic stages. In *PHOX2B∆8* mutant mice, we first confirmed faithful reporter GFP expression in all known Phox2b-expressing regions (e.g., hindbrain nuclei, enteric neurons) using co-labeling with an antibody for the unmutated N-terminal region of Phox2b protein (Figure S4b). No GFP expression was observed in WT littermates. Finally, we used IHC with an antibody for the C-terminal region of Phox2b protein (a region affected by *PHOX2B∆8* mutation) to confirm expression of the non-mutant allele in heterozygotes (not shown). These findings confirmed all expected characteristics of *PHOX2B∆8* expression in vivo.

Heterozygous *Hprt*-*cre*, *Phox2b∆8* pups showed perinatal lethality and died before P1. Harvest just prior to birth at embryonic day 18.5 (E18.5) revealed that only 33 % of mutants took one spontaneous breath (vs. 100 % in control, *n* = 8 mutants, 15 controls). No mutants showed further spontaneous respiratory effort; thus, all died within minutes of delivery. Electrophysiological recording from E18.5 ex vivo brain stem preparations showed depression of endogenous respiratory motor root output under baseline conditions and in response to the excitatory neuropeptide, substance P, confirming abnormal respiratory phenotype in *Hprt*-*cre*, *Phox2b∆8* mice (Fig. 1d), in keeping with other mouse models of CCHS [14, 26, 44, 54].

### Abnormal noradrenergic structures in brainstem of *Hprt*-*cre*, *Phox2b∆8* mice

**Fig. 3:**
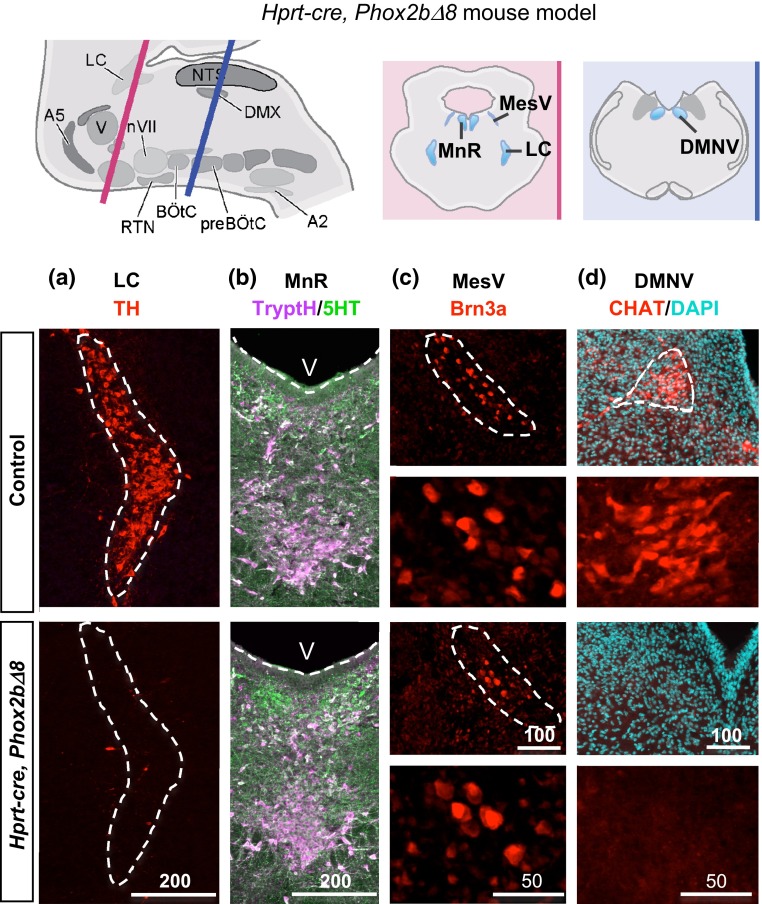
Brain pathology in *Hprt*-*cre*, *Phox2b∆8* mouse. *Top* Cartoon of mouse hindbrain at levels of rostral (*pink*) and caudal (*blue*) hindbrain is shown. **a**–**d** The *Hprt*-*cre*, *Phox2b∆8* mutant mouse showed profoundly abnormal differentiation of LC, characterized by absent expression of tyrosine hydroxylase (TH). Additional abnormalities were diminished MesV (Brn3) and DMNV Choline Acetyltransferase (CHAT) neurons. In contrast to NPARM *PHOX2BΔ8* proband (see Fig. 2b), the dorsal MnR in *Hprt*-*cre*, *Phox2b∆8* mouse showed non-significant reduction in counts of TrypH and 5HT cells compared with controls (*n* = 3). V, 4th ventricle. *Scale bar* *unit* µm

Generation of a patient-specific NPARM CCHS mouse model and findings from our human proband provided an opportunity for cross-species analysis to identify conserved neuropathological features (Table S1, Figs. 2, 3). In the mouse, we observed that the LC was also abnormal and failed to express tyrosine hydroxylase (TH), indicating a synthetic defect in noradrenergic pathway (Fig. 3a). Absence of TH neurons within the LC was correlated with sparse and small neuronal cell bodies, suggesting cellular loss/attrition rather than selective reduction of TH expression (Figure S5a). In addition, we observed consistent losses in the DMNV and mesencephalic trigeminal nucleus nuclei (MesV) (Fig. 3c, d). Neuronal precursors of the DMNV were detectable at E13.5 (Figure S5b) in the mouse model, suggesting *PHOX2B∆8* prevents DMNV formation despite progenitor specification. In contrast, while the dMnR showed severe attrition in the human proband (Fig. 2b), we found normal-appearing populations of serotonergic neurons that expressed tryptophan hydroxylase (17.00 ± 2.81 SEM (mutant) vs. 16.75 ± 2.79 SEM (control) cells per area, *p* = 0.812, *n* = 3) and 5HT (25.58 ± 9.13 vs 23.08 ± 1.88 cells per area, *p* = 0.952, *n* = 3, Student’s *t* test) spanning murine dMnR (Fig. 3b). No gross abnormalities in forebrain were observed. In summary, abnormal development of the LC was prominent consistently across species.

### PHOX2B∆8 inhibits LC noradrenergic neuronal specification

**Fig. 4:**
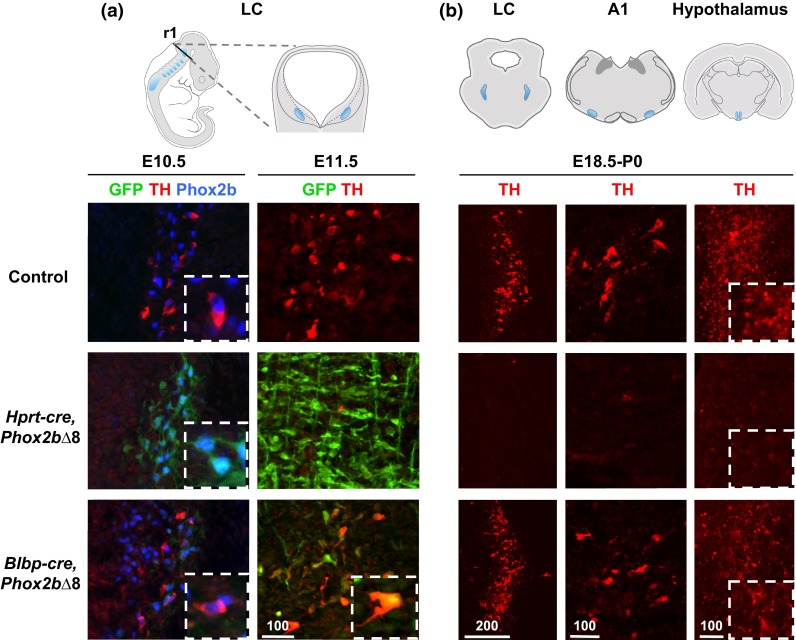
Noradrenergic neurons are affected by stage-specific activation of *PHOX2B∆8.* **a** *Left panels* the specification of noradrenergic neurons in LC appears by E10.5 in control, which express TH in addition to Phox2b. In *Hprt*-*cre* fate-mapped early-onset mice, the TH expression is diminished despite available Phox2b+ precursors. Note expression of PHOX2B∆8 protein (GFP+) in the same cells. *Blbp*-*cre* fate-mapped late-onset mice showed normal TH expressions and *PHOX2B∆8* is not expressed. *Right panels* by E11.5, cre-activation in late-onset mice commences in noradrenergic neurons, shown by GFP+ (*n* = 3–4). Note that mouse E10.5 and E11.5 are roughly equivalent to human gestational age week 6 and week 7, respectively. **b** Central noradrenergic neurons affected by early-onset *Hprt*-*cre*, *Phox2b∆8* were pervasive at E18.5 C-section including hindbrain nuclei LC and A1 and forebrain projection to periventricular nucleus of the hypothalamus. In the late-onset *Blbp*-*cre*, *Phox2b∆8,* noradrenergic neurons were intact in all areas at P0 (*n* = 3)

The LC is the major source of noradrenergic neurotransmitters in the CNS [5], and it projects to circuits in forebrain, midbrain and hindbrain [45]. As shown (Figs. 3a, 4b), early activation of *PHOX2B∆8* in the brainstem of *Hprt*-*cre*, *Phox2b∆8* mice resulted in developmental failure of TH + LC neurons. As we observed normal-sized populations of Phox2b-GFP+ precursors (Fig. 4a, discussed below), we conclude that early-onset *PHOX2B∆8* expression inhibits LC specification. Consistent with this, we observed widespread abnormalities in noradrenergic circuits, including caudal hindbrain nuclei A1/C2 and the forebrain projections of LC to hypothalamus (Fig. 4b, A1/C2 in Figure S5c).

Our conditional *Phox2b∆8* mouse model and cre-driver lines permitted introduction of *PHOX2B∆8* at two distinct time points in noradrenergic neuronal development. Whereas *Hprt*-*cre* introduces the mutation in the early embryo (<E10.5), *Blbp*-*cre* [23] results in CNS-restricted activation of the conditional *PHOX2B∆8* allele at E10.5 and later, after most neurogenesis. Fate mapping, using the conditional reporter function of the *PHOX2B∆8* mutant locus (Fig. 1c), showed robust onset of GFP expression in Phox2b+ cells at E10.5 with *Hprt*-*cre*, but only confined GFP+ population in *Blbp*-*cre* fate-mapped hindbrain (Figure S5d). In contrast, *Blbp*-*cre* targeting in brainstem was robust after E11.5 (Fig. 4a). Differences in prenatal viability between these two lines were noted (Figure S5e).

We next assessed consequences of early (<E10.5) versus later onset (>E11.5) of *PHOX2B∆8* expression for LC development. As shown (Fig. 4a), at E10.5 TH+, presumptive noradrenergic neurons were detectable in LC of control mice; moreover, these cells co-expressed Phox2b, consistent with previous findings [24]. Such TH+ populations were absent in the early-onset *Hprt*-*cre*, *Phox2b∆8* mice. Contrasting this, the late-onset *Blbp*-*cre*, *Phox2b∆8* model showed normal LC TH+ populations co-expressing Phox2b (Fig. 4a, b). The TH+, Phox2b+ populations did not express GFP, suggesting these cells expressed wild-type Phox2b allele. Together, these findings suggest that *PHOX2B∆8* inhibits LC noradrenergic differentiation in a stage-specific manner. That is, early-onset mutant protein expression derails LC differentiation in a dominant-toxic manner, whereas later-stage expression of the *PHOX2B∆8* allele does not interfere with acquisition of TH expression in Phox2b+-derived LC neurons.

### RTN development is uncoupled from the perinatal respiratory-lethal phenotype of *PHOX2B∆8* mice

**Fig. 5:**
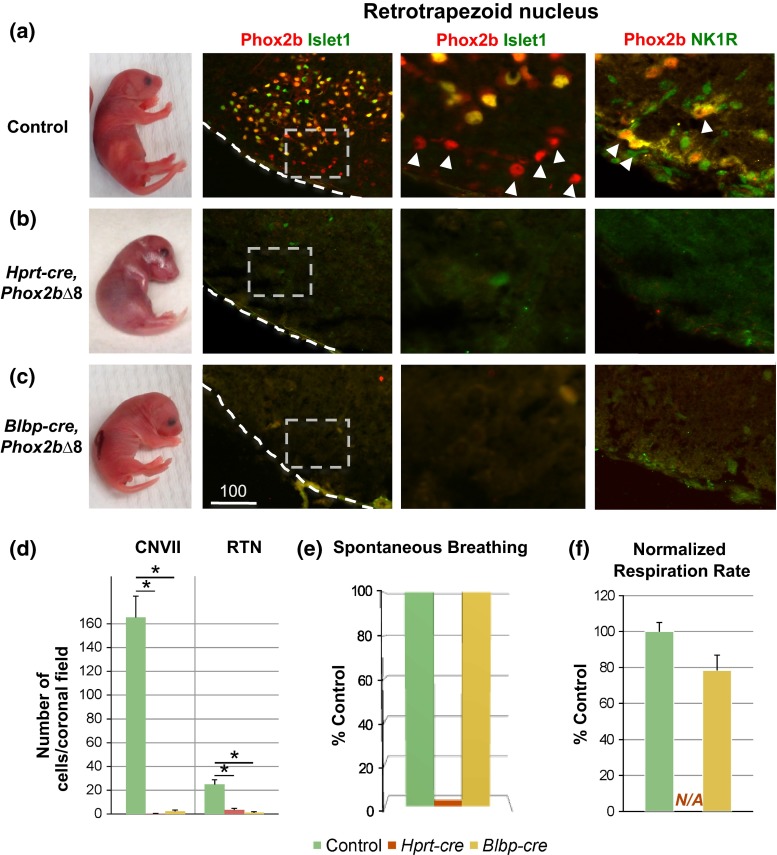
Central chemosensor RTN and CNVII are dispensable for perinatal respiratory regulation. **a**–**c** Respiratory phenotype and central chemosensor of **a** control, **b** early-onset *Hprt*-*cre*, *Phox2b∆8*, and **c** late-onset *Blbp*-*cre*, *Phox2b∆8* mice. Whereas *Hprt*-*cre*, *Phox2b∆8* mice failed to show any respiratory effort, were cyanotic (*dark violet skin tone*), and died in the immediate perinatal period, *Blbp*-*cre*, *Phox2b∆8* mice showed spontaneous respiration and were *pink* (*n* = 7–11 from at least 3 litters/genotype). Both *Hprt*-*cre*, *Phox2b∆8* and *Blbp*-*cre*, *Phox2b∆8* mice lacked the central chemosensor RTN (Phox2b+/Islet−, and NK1R+/Phox2b+) and CNVII (Phox2b+/Islet+), despite continuous respiration in *Blbp*-*cre*, *Phox2b∆8* and lack of respiration in *Hprt*-*cre*, *Phox2b∆8* (*n* = 3). **d** Quantification of RTN (Phox2b+/Islet−) and CNVII (Phox2b+/Islet+) showed significant losses of both nuclei at E18.5 (*n* = 3). **e** When mice were harvested at E18.5, no *Hprt*-*cre*, *Phox2b∆8* mice showed continuous respiration in contrast to *Blbp*-*cre*, *Phox2b∆8* and control mice (*n* = 7–11 from at least 3 litters/genotype).** f** Quantification of breathing rate at birth showed spontaneous/continuous breathing in *Blbp*-*cre*, *Phox2b∆8* mice albeit at reduced frequency. Respiratory rate ranged 20–132 breaths/min depending on time post-birth, normalized to control within the same litter (*n* = 3–5). *N/A* not assessed due to absence of respiration

We observed striking differences in the perinatal respiratory phenotype of early-onset *Hprt*-*cre*, *Phox2b∆8* versus late-onset *Blbp*-*cre*, *Phox2b∆8* models (Fig. 5). As mentioned above, *Hprt*-*cre*, *Phox2b∆8* animals showed little/no spontaneous respiratory effort, rapidly became cyanotic (dark violet skin tone) and died minutes after birth (Fig. 5b, e–f). In contrast, *Blbp*-*cre*, *Phox2b∆8* mice were typically pink (non-cyanotic) and showed spontaneous/continuous breathing (albeit at slightly reduced frequency; 78 ± 8.5 % of control respiratory rate, *n* = 3, *p* > 0.1, Student’s *t* test) (Fig. 5c, e–f). Longitudinal observation of *Blbp*-*cre, Phox2b∆8* mice past P1 was not possible, as the mutants failed to nurse and gain adequate body weight.

**Fig. 6:**
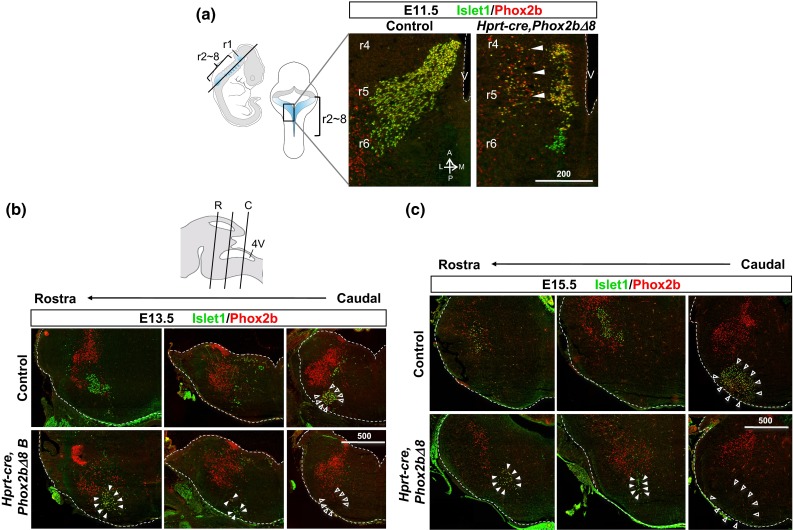
Abnormal migration of facial nucleus CNVII in *Hprt*-*cre*, *Phox2b∆8* mouse. **a** Neurons of CNVII migrate caudally from their origin in rhombomere (r)4 toward r6 followed by lateral migration toward ventral surface. Horizontal sections at E11.5 to visualize trans-rhombomere migration showed in *Hprt*-*cre*, *Phox2b∆8* mouse, precocious termination of rostral-to-caudal migration (note accumulation of Phox2b+ Islet1+ cells at r4–5), and abnormal lateral migration within r4–5 (*arrowheads*) (*V* 4th ventricle, *A* anterior, *P* posterior, *L* lateral, *M* medial). **b** *Top* cartoon of mouse hindbrain levels of rostral (R) and caudal (C) at E14 is shown. At E13.5, stalled migration of CNVII/RTN, detected by Islet1 and Phox2b antibodies, was found at rostral levels (denoted by *solid arrowheads*) of hindbrain than normally found at caudal level (denoted by *empty arrowheads*) in control littermates. **c** The same pattern described in **b** was observed at E15.5, implicating permanent migration defect of CNVII. The number of putative Phox2b+ Islet1+ CNVII cells found in the mutants declined from 37.6 to 16.2 % of WT at E13.5 and E15.5, respectively. *Scale bar unit* µm

One possibility to account for these differences was differential effects of early versus late *Phox2b∆8* expression on RTN development [34]. However, histological examination of the brainstems from both *Hprt*-*cre*- and *Blbp*-*cre*-driven models showed loss of RTN and CNVII nuclei, as shown by IHC and quantitative analysis of the markers Phox2b, Islet1 and neurokinin 1 receptor (NK1R) (Fig. 5a–d). Moreover, developmental analysis of embryonic brainstem confirmed that abnormal formation of CNVII was due to failure of precursor migration in the *Hprt*-*cre, Phox2b∆8* mouse model (Fig. 6a–c), in keeping with reported findings in other CCHS mouse models [10, 13, 26, 35]. A similarly mis-located putative CNVII was found in *Blbp*-*cre, Phox2b∆8* mouse (Figure S5f). Analysis of MnR in *Blbp*-*cre, Phox2b∆8* mouse showed normal number of serotonergic neurons expressing TryptH (17.833 ± 1.815 SEM cells per area, *p* = 0.682, *n* = 3, and 5HT (Figure S5g) (27.166 ± 1.249 SEM cells per area, *p* = 0.793, *n* = 3, Student’s *t* test). Thus, our findings in late-onset *Blbp*-*cre, Phox2b∆8* mice indicate that the RTN is dispensable for generation of minimal perinatal respiratory rhythm, in keeping with the proposal that self-evoked respiration is possible without the RTN [26, 44].

### Human CCHS proband 2: clinical history and neuropathological findings

Proband 2 was a 27 5/7-week gestation age preterm infant male. He was intubated at birth due to poor respiratory effort and received surfactant. Although there was no evidence for chronic lung disease of prematurity, the patient remained ventilator dependent. At approximately 4 weeks of age, lack of respiratory drive despite persistent hypercapnia prompted genetic testing for CCHS. Proband 2 carried a heterozygous PARM *PHOX2B* mutation, resulting in the most common, 7-residue alanine expansion (*PHOX2B* 20/27 genotype). After withdrawal of life support at ~41-week corrected gestational age, autopsy was performed with postmortem analysis of brainstem.

While the pons contained a cluster of cells morphologically and anatomically consistent with the LC, as shown in Figs. 2c and S6, they failed to express normal levels of DBH or TH, indicating LC dysfunction and noradrenergic synthesis. We did not observe gliosis or other signs indicative of hypoxic damage in the brain stem. In contrast to the NPARM *PHOX2BΔ8* CCHS proband 1 (Fig. 2b), the MnR, MesV, and DMNV hindbrain nuclei in the PARM subject appeared normal (Figure S6). Taken together, our findings indicate that both NPARM and PARM *PHOX2B* mutations result in defects of LC neuronal populations in human neonates with CCHS.

## Discussion

The underlying pathobiology of CCHS remains unclear, reflecting in part the complexity of central and peripheral centers that interact to control respiratory drive. We used a combinatorial approach incorporating human neuropathological analysis from two (NPARM and PARM) human probands and a conditionally activated NPARM patient-specific transgenic mouse model to study temporal effects of mutant protein on brainstem development. Our study is the first to describe CNS neuropathological findings in two human cases of CCHS with confirmed PARM and NPARM mutations of *PHOXB2B,* which are summarized in Table S1. Further, we modeled the proband-specific NPARM mutation introduced at several stages of mouse hindbrain development. While several structures are affected variously in these cases, a focus on conserved abnormalities between species revealed abnormalities in LC populations, which probably result from failure to specify LC neurons in the embryonic brain. Our findings suggest that disruption of LC noradrenergic neuron development and function may be a common pathobiological feature of CCHS.

### Brainstem pathological findings in two human CCHS cases with confirmed NPARM and PARM *PHOX2B* mutations

The LC is the major source of noradrenergic innervation to both rostral brain regions as well as the brainstem. Abnormal noradrenergic signaling has been implicated in clinical features of CCHS [32, 55]. Studies of CCHS by diffusion MRI (albeit without confirmed *PHOX2B* mutations) showed altered diffusivity (decreased fractional anisotropy and increased axial and radial diffusivity) in several brainstem regions as well as other potentially connected regions of the mid-hindbrain [41]. Such late structure/function studies in adolescents carry the caveat that injury to LC or dMnR could have accrued from cumulative effects of repeated episodes of hypoxemia.

In contrast, the two NPARM and PARM probands we studied were neonates that were intubated and/or managed in an NICU from birth with stringent monitoring and interventions to prevent hypoxemic events after birth. Proband 1 carried a NPARM mutation of (*PHOX2B∆8)* and demonstrated severe CCHS and intestinal aganglionosis (Haddad syndrome). Postmortem analysis showed several brainstem nuclei were abnormal (Table S1) including almost total absence of LC neurons. Furthermore, a second *PHOX2B 20/27* PARM proband 2 also showed defects in DBH and TH expression in the context of a well-formed LC, indicating deficient numbers of functioning noradrenergic neurons. The correspondence of abnormalities in the LC in both human cases is striking. While further confirmation in additional cases of CCHS would be useful, such pathological specimens in confirmed cases of neonatal CCHS with *PHOX2B* mutation are extremely rare. We note these data are consistent with a previously reported case of CCHS (albeit without a confirmed *PHOX2B* mutation), showing significant defects in noradrenergic cell number [52]. Together, these findings suggest that abnormal development of the LC is common in CCHS.

### NPARM *PHOX2B∆8* permits brainstem noradrenergic neuron precursor allocation but inhibits differentiation in a stage-restricted manner

Our findings demonstrate that inhibitory effects of PHOX2B∆8 proteins during LC development are stage restricted. The murine LC is formed during E9–E11 [50] and by E11.5 it is a clearly identifiable structure [3]. Human equivalent developmental stage for mesencephalic TH neurons occurs 6.5–8 weeks post-conception [16, 36]. We found that early mutant protein expression (using *Hprt*-*cre*) prevented LC neuron specification/differentiation to a TH+ state. In contrast, delayed expression of the mutation (with *Blbp*-*cre*) permitted LC neuron differentiation to the TH+ stage. Together, these findings indicate *PHOX2B∆*8 proteins inhibit early LC neuronal specification, rather than the program of expression characteristic of mature LC neurons.

Abnormal respiratory arousal during NREM sleep is associated with dysregulation of central adrenergic [38] and serotonergic [25, 43] signaling. Caudally, the LC densely innervates the serotonergic dorsal raphe nucleus [30]. The dorsal raphe nucleus does not express PHOX2B. Therefore, the finding in human proband 1 that the dorsal raphe was lost is consistent with the possibility of long-term failure of normal feedback mechanisms. For example, classical ultrastructural studies have demonstrated in experimental animals innervation of serotonergic neurons by noradrenergic locus coeruleus neurons [4]. This circuitry between noradrenergic and serotonergic systems raise the possibility that disease affecting the noradrenergic system could cause secondary effects to the serotonergic neurons through transneuronal degeneration mechanisms similar to neurodegenerative diseases [15]. In keeping with this possibility, the NPARM early-onset mouse model did not show defects in the dMnR. Moreover, we observed that noradrenergic circuit formation in hypothalamus and A1/C2 nuclei of brain stem were also rescued in the *Blbp*-*cre, Phox2b∆8* mice, suggesting that PHOX2B∆8 generally exerts its impact on noradrenergic neuron development at an early stage. Further work is needed to identify precise gene targets affected in the early-onset phenotype (see discussion below).

### Evidence that RTN is dispensable for perinatal respiratory drive in the NPARM CCHS model

The RTN is generally thought to have critical roles in perinatal respiratory control in rodents [21]. However, while conditional targeting of *Phox2b* function resulted in failure of RTN development and lethal respiratory compromise in one study [14], another study that selectively targeted disruption of the RTN with *Egr2*-*cre* did not cause perinatal respiratory lethality and suggested its importance might be specific to chemosensation [44]. We found that while late-onset *PHOX2B∆8* expression caused failure of RTN and CNVII development, animals showed near-normal perinatal respiration, indicating dispensability of the RTN for this function. Unfortunately, such *Blbp*-*cre, PHOX2B∆8* animals did not survive past P1 due to oropharyngeal problems preventing feeding and so further testing was not performed. Nevertheless, our studies suggest the RTN is dispensable as an early regulator of respiratory drive. In the human, the equivalent structure to RTN has been suggested [29, 47], but its existence remains controversial. Despite exhaustive efforts, we failed to identify an RTN-like structure in our proband cases or specimens from five age-matched unaffected subjects, and the facial nucleus (CNVII) was normal in appearance in the human *PHOX2BΔ8* proband (Figure S3b).

### Dysregulation of locus coeruleus development might be general feature of human CCHS

Understanding mechanisms that underlie CCHS has general implications for development of human respiratory control [22] and other disorders of respiratory and autonomic regulation including Rett Syndrome [37], sudden infant death syndrome (SIDS) and apnea of prematurity. Findings from two human CCHS cases indicate that NPARM and PARM *PHOX2B* mutations disrupt development of LC noradrenergic populations, a finding that is phenocopied in the NPARM CCHS mouse model we generated, but not in another previously reported mouse model of *PHOX2B 20/27* PARM [13], which failed to show defects in the LC. Why the mouse *PHOX2B 20/27* model fails to capture aberrant LC neuron differentiation is unclear, but might reflect differences between mouse and human development in the context of 20/27 PARM mutations.

Several lines of evidence support the role of LC as a central chemosensor [17]. First, *Phox2a* function is required for differentiation of LC but leaves other noradrenergic centers (locus subcoeruleus and groups A7, A5, A2 and A1) intact, and loss of *Phox2a* function results in depressed central respiratory drive [60]. Thus, it is possible that mutant PHOX2B proteins act in a “dominant-negative” fashion to inhibit LC neuron specification. Second, loss of the LC is associated with markedly decreased breathing frequency [56]. Third, in a mouse model of Rett syndrome, caused by mutations of methyl-CpG-binding protein 2 (MECP2), there is loss of LC neurons [46, 51], and breathing dysfunction with decreased CO_2_ chemosensitivity [61]. Together, these findings suggest that LC dysfunction might explain, at least in part, central CO_2_ chemo-insensitivity in CCHS as well as failure of normal respiratory arousal. Further research is needed to evaluate the utility of pharmacological approaches that might target noradrenergic signaling imbalance so that the deleterious effects of *PHOX2B* mutations on CCHS patients are attenuated. Indeed, maturational decrement in ventilatory slope in response to hypercarbia/hypoxia is observed in CCHS [8] suggesting that early pharmacologic noradrenergic stimulation might assuage disease progression.

## Electronic supplementary material

Supplementary material 1 (DOC 61 kb)

Supplementary material 2 (PPTX 186,429 kb)
