# Supplementary material for: Dysregulation of locus coeruleus development in congenital central hypoventilation syndrome
Source: Acta Neuropathol. 2015 May 15;130(2):171–83. doi: 10.1007/s00401-015-1441-0 (PMC4503865; doi:10.1007/s00401-015-1441-0)
Supplement: Supplementary file 1 — Supplementary material 1 (DOC 61 kb) [file 401_2015_1441_MOESM1_ESM.doc]

**Supplementary Data**

|  | Title |
| --- | --- |
| Table S1 | Summary of pathological findings in human CCHS probands and transgenic mouse models. |
| Figure S1 | The original, entire 120-second epoch from the polysomnographic recording of the NPARM *PHOX2BΔ8* CCHS proband. |
| Figure S2 | Loss of enteric neurons in human NPARM *PHOX2BΔ8* CCHS proband and *Hprt-cre*, *Phox2b∆8* mouse. |
| Figure S3 | Pertinent genetic and neuropathological findings in NPARM *PHOX2BΔ8* CCHS proband. |
| Figure S4 | Generation of patient-specific, inducible *Phox2b∆8* transgenic mouse. |
| Figure S5 | Pertinent findings in *Phox2b∆8* mouse model. |
| Figure S6 | Immunohistochemical characterization of CCHS PARM and NPARM Neuropathology |

**Table S1.** Summary of pathological findings in human CCHS probands and transgenic mouse models. CNVII; facial nucleus, RTN; retrotrapezoid nucleus, nTS; solitary nucleus, MesV; mesencephalic trigeminal nucleus, DMNV; dorsal motor nucleus of the vagus, LC; locus coeruleus, A1/A5; A1 and A5 noradrenergic nuclei. + indicates pathology detected, - indicates pathology not detected. N/D not done.

**Figure S1**. The raw, entire 120-second epoch from the polysomnographic recording of the NPARM *PHOX2BΔ8* CCHS proband without respiratory support.

The polysomnographic recording performed with CPAP 5cm H_2_O during NREM sleep stage 1 and 2 (N1, N2) demonstrates hypoventilation (low amplitude chest and abdomen respiratory efforts) with ineffective breathing and lack of increase of respiratory effort despite persistently elevated end-tidal carbon dioxide concentration (E_T_CO_2_) and decreased oxygen in the circulating blood (SpO_2_). The entire 120-second segment is shown. Four EEG channels (C3-02; C4-O1; O1-FZ; O2-FZ) were used to monitor the sleep stages and demonstrate the lack of arousal response to hypercarbia and hypoxemia.

**Figure S2.** Loss of enteric neurons in human NPARM *PHOX2BΔ8* CCHS proband and *Hprt-cre*, *Phox2b∆8* mouse.

Diagram of human intestine is shown for orientation. In the human proband, the tissue section of small intestine, taken 10 cm distal to the Ligament of Treitz, shows near absence of neuronal marker synaptophysin. In *Hprt-cre*, *Phox2b∆8* mouse, a similar loss of neuronal marker Tuj1 was observed in the mutant intestine at E18.5.

**Figure S3.** Pertinent genetic and neuropathological findings in NPARM *PHOX2BΔ8* CCHS proband.

a. Predicted amino acid sequence of the proband. The mutation caused an aberrant sequence starting amino acid position 230 (asterisk) and elongation beyond wildtype 214 amino acid length (arrowhead).

b. The proband’s hindbrain sections showed arcuate nucleus, facial nucleus or surrounding areas, inferior olive, area postrema and nucleus prepositus were without morphological abnormality.

**Figure S4.** Generation of patient-specific, inducible *Phox2b∆8* transgenic mouse and confirmation of faithful mutant expression.

a. The targeting construct is shown in (1). Wildtype mouse *Phox2b* gene contains 3 exons. Exon3 is flanked by *loxP* sites for Cre-mediated conditional expression. This construct is followed by human exon3, which contains patient-specific mutation (denoted as “8del” for 8 base-pair deletion) in addition to eGFP reporter construct. Note human and mouse exon 3 are identical at the amino acid level but not nucleotide base level; use of human exon 3 in place for mouse exon 3 allows expression of the mutant amino acid sequence in mouse. This targeting vector was used to replace a wildtype allele shown in (2) via homologous recombination to create heterozygous insertion of target construct shown as (3). Neomycin resistant gene contained in target construct was used to select for correct recombination, followed by removal of gene by Flp recombinase, resulting in the final transgenic gene structure in (4).

b. Faithful expression of mutant protein and eGFP reporter was confirmed in the mutant mouse generated by crosses with germline *Hprt*-cre driver by colocalization of eGFP to known populations of Phox2b-dependent hindnbrain neurons during embryonic development at E11.5 (migrating rhombomere neurons) and E18.5 (nTS; solitary nucleus).

**Figure S5.** Pertinent findings in *Phox2b∆8* mouse model.

a. In the LC, decreased density of NeuN+ terminally mature neurons and lack of TH expression was observed despite numerous reporter (GFP) expressions in the same area. GFP+ cells showed dim NeuN expression (empty arrowheads), and only rarely TH+ cells showed GFP (solid arrowhead).

b. Precursors of the dorsal motor nucleus of vagus (DMNV), found to be lost in *Hprt-cre*, *Phox2b∆8* mouse at E18.5, was detectable at E13.5, suggesting failure of survival.

c. The loss of noradrenergic neurons in the central nervous system extended to caudal hindbrain nuclei including A1 and C2 in *Hprt-cre*, *Phox2b∆8 mice*.

d. Blbp-cre driver induced expression of GFP+ Phox2b∆8 in restricted hindbrain regions at E10.5. (V = 4^th^ ventricle).

e. Hprt-cre, Phox2b∆8 litters at E11.5 typically showed a few resorbed embryos (arrows) and decreased viability at P0 (36% live born observed versus 50% expected). In contrast, E11.5 Blbp-cre, Phox2b∆8 litters showed normal survival in Mendelian ratios. Mutants are identified by asterisks.

f. A mislocated putative CNVII, detected by Islet1 and Phox2b antibodies was found at rostral levels (denoted by solid arrowheads) of hindbrain in Blbp-cre, Phox2b∆8 mice in contrast to normally found caudal level (denoted by empty arrowheads) in controls.

g. The dorsal MnR in Blbp-cre, *Phox2b∆8* mouse showed non-significant difference in counts of TrypH and 5HT cells compared to controls. V, 4^th^ ventricle.

**Figure S6.** Immunohistochemical charachterization of CCHS PARM and NPARM Neuropathology.

a. Cartoon of human hindbrain at levels of pons (pink) and medulla (blue) are shown on top.

b. PARM proband 2 was compared to a case control (term infant that expired with hypoxic ischemic encephalopathy). Morphological and immunohistochemical evaluation was performed on the locus ceruleus (LC), median raphe (MnR), mesencephalic trigeminal nucleus (MesV), and the dorsal motor nucleus of the vagus nerve (DMNV). Reduced expression of tyrosine hydroxylase (TH) and neurons with low intensity staining was noted in LC in the CCHS PARM patient (arrowheads), consistent with DBH immunohistochemistry (Figure 2). HE& staining showed no reduction in neuronal number. Preserved tryptophan hydroxylase (TryptH) and choline acetyle transferase (CHAT) were noted in the MnR and DMNV, respectively. (H&E = hematoxylin and eosin).

c. H&E staining of LC in NPARM proband 1, demonstrating reduced neuronal number.

**Supplementary Methods**

**Case histories of controls:**

For the NPARM proband (proband 1), control case 1 was a full term 8-week old female with hypoplastic left heart syndrome status post cardiac surgery. Case 2 was a full term 1-day old male with mid-gut volvulus and near-total necrosis of the small intestine. Case 3 was a full term 12-week old with congenital diaphragmatic hernia and severe hypoxic ischemic encephalopathy. Case 4 was a full term 5-day old female with severe hypoxic ischemic encephalopathy. For the PARM proband (proband 2), the control case was a 5-day old female with 41 and 5/7 gestational age, diagnosed with hypoxic ischemic encephalopathy.

**Human neuropathological studies:**

Antibodies for human immunohistochemical (IHC) studies employed: Tryptophan hydroxylase (Sigma T0678, 1:1000), synaptophysin (DAKO clone SY38, 1:50), dopamine b-hydroxylase (DBH) (Sana Cruz SC15318, 1:100), tyrosine hydroxylase (TH) (chemicon AB152, 1:500), Choline Acetyltransferase (Millipore AB144P, 1:100), and MAP2 (Cell Signaling 4542S, 1:50). Antibodies were developed using DAKO envision kits (K4004 and K4011). Antigen retrieval was performed by incubation in Citrate Buffer, pH 6.0 for 25 minute at 95° C in a Pelco BioWave Pro microwave. A standard neuroanatomy brain atlas was used to guide the comparisons .

**DNA sequencing:**

DNA sequencing was performed by Ambry Genetics. Confirmation of *PHOX2B* sequence from proband- and control fibroblasts was conducted by PCR (Invitrogen, AccuprimeTM GC-Rich DNA Polymerase kit), using the following primers. Forward primer: 5’- GCCAAGTTTCGCAAGCAGGAGCGCG -3’. Reverse primer: 5’-

CTTCACTAAGGCGGCTTTGGCACCGTTGGG-3’. These amplify a 480 base pair fragment of *PHOX2B* exon 3.

**Mouse tissue histology:**

Primary antibodies for mouse histology included N-terminal PHOX2B (Santa Cruz Biotechnology, sc-1322, 1:200), Tyrosine hydroxylase (Millipore, AB152, 1:1000), Islet1/2 (DSHB, 39.4D5, 1:20), Tuj1 (Covance, MMS-435P, 1:1000), GFP (Aveslab, GFP-1020, 1:1000), Choline Acetyltransferase (Millipore, AB144P, 1:100), Tryptophan hydroxylase (Abcam, Ab3907, 1:500), 5HT (Immunostar, 20080, 1:2000). Secondary antibodies were purchased from Invitrogen or Jackson ImmunoResearch Laboratories.

***In vitro* mouse explant respiratory physiology:**

For brainstem-spinal preparation, E18.5 embryos were delivered under anesthesia (ketamine/xylazine mixture) by cesarean section from timed-pregnant female mice. The dissections was done while keeping the embryos submerged in cold (4°C) artificial cerebral spinal fluid [regular/ enhanced aCSF (in mM): 124 NaCl, 3/5 KCl, 1.5/2.4 CaCl_2_, 1.0/ 1.3 MgSO_4_, 25.0/ 26.0 NaHCO_3_, 0.5 NaH_2_PO_4_/ 1.2 KH_2_PO_4_, 30 D-Glucose (Sigma) equilibrated with 95% O_2_ and 5% CO_2_ to pH = 7.4] and transferred into a partitioned 6 ml recording chamber, which were separately gravity fed by reservoirs of heated (25°C–26°C) and aerated (95% O_2_ and 5% CO_2_) aCSF at a rate of 3–4 ml/min.

**References Cited:**

1 DeArmond SJ, Fusco MM, Dwewey MM (1989) Structure of the Human Brain, A Photographic Atlas. Oxford University Press, City
